# Training physicians in India to interpret pediatric chest radiographs according to World Health Organization research methodology

**DOI:** 10.1007/s00247-021-04992-2

**Published:** 2021-03-11

**Authors:** Eric D. McCollum, Melissa M. Higdon, Nicholas S. S. Fancourt, Jack Sternal, William Checkley, John De Campo, Anita Shet

**Affiliations:** 1 Department of International Health, Johns Hopkins Bloomberg School of Public Health, Baltimore, MD USA; 2 Department of Pediatrics, Johns Hopkins Global Program in Respiratory Sciences, Eudowood Division of Pediatric Respiratory Sciences, Johns Hopkins School of Medicine, 200 North Wolfe St., Baltimore, MD 21287 USA; 3 Department of International Health, International Vaccine Access Center, Johns Hopkins Bloomberg School of Public Health, Baltimore, MD USA; 4 Global and Tropical Health Division, Menzies School of Health Research, Charles Darwin University, Darwin, Australia; 5 Murdoch Children’s Research Institute, Melbourne, Australia; 6 Division of Pulmonary and Critical Care, School of Medicine, Johns Hopkins University, Baltimore, MD USA; 7 Center for Global Non-Communicable Disease Research and Training, School of Medicine, Johns Hopkins University, Baltimore, MD USA; 8 Department of Radiology, Melbourne University, Melbourne, Australia

**Keywords:** Asia, Chest, Child, India, Infant, Pneumonia, Radiography, Respiratory tract, World Health Organization

## Abstract

**Background:**

Chest radiography is the standard for diagnosing pediatric lower respiratory infections in low-income and middle-income countries. A method for interpreting pediatric chest radiographs for research endpoints was recently updated by the World Health Organization (WHO) Chest Radiography in Epidemiological Studies project. Research in India required training local physicians to interpret chest radiographs following the WHO method.

**Objective:**

To describe the methodology for training Indian physicians and evaluate the training’s effectiveness.

**Materials and methods:**

Twenty-nine physicians (15 radiologists and 14 pediatricians) from India were trained by two WHO Chest Radiography in Epidemiological Studies members over 3 days in May 2019. Training materials were adapted from WHO Chest Radiography in Epidemiological Studies resources. Participants followed WHO methodology to interpret 60 unique chest radiographs before and after the training. Participants needed to correctly classify ≥80% of radiographs for primary endpoint pneumonia on the post-training test to be certified to interpret research images. We analyzed participant performance on both examinations.

**Results:**

Twenty-six of 29 participants (89.7%) completed both examinations. The average score increased by 9.6% (95% confidence interval [CI] 5.0–14.1%) between examinations (*P*<0.001). Participants correctly classifying ≥80% of images for primary endpoint pneumonia increased from 69.2% (18/26) on the pretraining to 92.3% (24/26) on the post-training examination (*P*=0.003). The mean scores of radiologists and pediatricians on the post-training examination were not statistically different (*P*=0.43).

**Conclusion:**

Our results demonstrate this training approach using revised WHO definitions and tools was successful, and that non-radiologists can learn to apply these methods as effectively as radiologists. Such capacity strengthening is important for enabling research to support national policy decision-making in these settings. We recommend future research incorporating WHO chest radiograph methodology to consider modelling trainings after this approach.

**Supplementary Information:**

The online version contains supplementary material available at 10.1007/s00247-021-04992-2.

## Introduction

According to 2017 Global Burden of Disease estimates, one to two children worldwide die every minute before their fifth birthday from pneumonia [1]. Nearly 200,000 children in India — more than in any other country — perish from lower respiratory infections each year [1, 2]. Among lower respiratory infections, bacterial pneumonia attributable to *Streptococcus pneumoniae* is a significant cause of death among those younger than 5 years old in India and other low-income and middle-income countries, as well as globally [3, 4]. Introducing pneumococcal conjugate vaccines and lowering household air pollution together reduced global lower respiratory infection mortality by about 15% between 1990 and 2017 [1]. India recently introduced the pneumococcal conjugate vaccine into its Universal Immunization Program with assistance from Gavi (a public-private effort for equitable vaccine access globally) [5], and the government has continued to support liquified petroleum gas subsidy programs to combat household air pollution [6].

The World Health Organization (WHO) methodology for classifying pediatric chest radiographs, developed in 1997, has been applied to efficacy and effectiveness studies of bacterial conjugate vaccines as well as more recently to epidemiological research of pediatric lower respiratory infections [7–16]. This methodology aims to identify chest radiographs that have a higher probability of bacterial etiology, with an emphasis on *Streptococcus pneumoniae* and *Haemophilus influenzae*, and prioritizes both interobserver reliability and specificity [8, 15]. Interpretations based on this method are for research only and not intended for clinical use [15]. To assist researchers using the WHO methodology, the WHO Chest Radiography in Epidemiological Studies project was launched in 2016 to clarify standardized definitions and develop open-access training and support tools [17].

A chest radiograph reading panel was established to support two research projects in India that aimed to evaluate pneumococcal conjugate vaccine effectiveness and estimate liquified petroleum gas efficacy against child pneumonia [18, 19]. The revised WHO Chest Radiography in Epidemiological Studies definitions and training and support tools were adapted for a 3-day training in Agra, India, during May 2019. Our objectives for this research were twofold. First, we sought to describe the methodology and share the materials used to train chest radiograph readers in India for these projects. Second, we aimed to evaluate the effectiveness of the training program.

## Materials and methods

### World Health Organization chest radiograph interpretation training

Twenty-nine physicians, all of whom were practicing throughout India, participated in the training held May 3–5, 2019. Fifteen of the physicians were radiologists and 14 were pediatricians. We sought a geographically representative expert reading panel with strong local leadership capacity. Thus, participants were recruited by targeted inquiries taking into account both their geographic location within India and giving priority to those having leadership positions at reputed Indian medical institutions. To provide participants context for the meeting, we shared optional background reading materials a week before the training. Participants were encouraged but not required to complete the readings [8, 17].

The training objectives were threefold: (1) to achieve predetermined certification requirements using the WHO methodology (described below), (2) to understand the technical requirements and expected workflow for the research projects, and (3) to discuss quality assurance procedures for the research projects. To accommodate adult participants with a wide variety of medical backgrounds, professional experience and learning styles, the overall training approach sought to be participatory and active, with a mixture of large and small group sessions and also individual sessions. All sessions were co-facilitated by WHO Chest Radiography in Epidemiological Studies members (E.D.M., J.D.C.).

### Pretraining examination

All participants completed a pretraining examination after listening to introductory presentations on the overall background and goals of the associated research projects. Sixty WHO Chest Radiography in Epidemiological Studies and WHO reference chest radiographs, previously adjudicated by a panel of experts and determined to have high agreement amongst the expert panel for findings of interest, including either primary endpoint pneumonia, other infiltrate, neither, or uninterpretable, comprised the pretraining examination. High agreement was defined in the WHO Chest Radiography in Epidemiological Studies set of images as >66% agreements among an expert panel, while in the WHO reference images this was not explicitly defined. Sixty images were selected based on balancing the desire to have a representative distribution of image classifications and a reasonable examination length. Please see Table 1 for the WHO radiologic definitions. In order to be consistent with the post-test examination — where the goal was to test participants on their ability to correctly identify images with and without primary endpoint pneumonia — we intentionally selected an image distribution for each test of which approximately half of the images were positive for primary endpoint pneumonia, meeting this classification based on a variety of features (i.e. silhouette sign, pleural effusion or endpoint consolidation). Test participants were permitted to refer to decision-support tools designed by E.D.M. but not yet introduced formally to the participants. Participants completed the pretraining examination individually. The examination format allowed participants 1 minute to classify each image. We displayed chest radiograph images on a projection screen in a darkened room, but participants could also view images or their laptop screen if preferred. We asked readers to provide interpretations on the classification of findings for each hemithorax and for the overall image quality, according to Table 1. Conclusions were derived from program logic based on participants’ responses to each question on the assessment form (Table 2) and in accordance with the criteria in Table 1, and the reader confirmed this for each image. For example, if a participant selected that the image had an endpoint consolidation and an other infiltrate (regardless of which hemithorax) then the system would prompt the participant to confirm that their final classification was “primary endpoint pneumonia with an other infiltrate.” Both our examination and teaching emphasis were, therefore, focused on identifying or excluding endpoint consolidation, silhouette sign and pleural effusion and how these features related to the main primary endpoint pneumonia classification. The counting of anterior and posterior ribs was only mandated on 10% of images in the pretraining examination and was not considered in the scoring of the pretraining test.

**Table 1:** Definitions for World Health Organization chest radiograph interpretation research methodology [17]

Quality	Uninterpretable	Image is not interpretable regarding the presence or absence of endpoint consolidation or pleural effusion without repeat imaging.
	Suboptimal	Image interpretable for endpoint consolidation and pleural effusion but not other infiltrate.
	Adequate	Image allows confident interpretation of all features.
Classification of findings	Significant pathology	Presence of endpoint consolidation, other infiltrates or pleural effusion.
	Endpoint consolidation	An opacity that includes a portion^a^ or whole of a lobe, or the entire lung, that is dense or fluffy in appearance and may or may not contain air bronchograms^b^.
	Other infiltrate	Densities that appear linear, patchy and lacy (interstitial infiltrate), including peribronchial thickening and atelectasis; can also be smaller patchy infiltrates or atelectasis that do not meet the criteria of endpoint consolidation.
	Pleural effusion	Fluid in the lateral pleural space either at the costophrenic angle or adjacent to the lateral chest wall that is spatially associated with an opacity (either endpoint consolidation or other infiltrate) or is of large enough size in the hemithorax that an opacity may be obscured; not including fluid in the horizontal or oblique fissures.
Conclusions	Primary endpoint pneumonia	Presence of endpoint consolidation or pleural effusion (as defined).
	Other infiltrate	Presence of other infiltrate (as defined) in the absence of pleural effusion.
	No consolidation, infiltrate or effusion	No endpoint consolidation, other infiltrate or pleural effusion.

^a^“Portion” indicates an objective size dimension for an opacity, defined as an opacity’s smallest diameter greater than or equal to the size of a posterior rib and one adjacent rib space at the same level as the opacity. For an irregularly shaped opacity (e.g., wedge-shaped), use the maximum short-axis diameter (largest diameter perpendicular to the line of maximum diameter of the opacity)

^b^An opacity that creates a silhouette sign, defined as the loss of an anatomical border greater than or equal to the size of a posterior rib and one adjacent rib space at the same level, is an endpoint consolidation. A silhouette sign of this length but without a visible adjacent opacity is an other infiltrate

**Table 2:** Chest radiograph (CXR) assessment

Are any of the following present?	
1. **Endpoint consolidation** (*an opacity that BOTH meets the size criteria [1 rib+1 rib space] AND is sufficiently dense [±air bronchogram]*) a. Patient right side: yes/no/unable to assess due to CXR quality b. Patient left side: yes/no/unable to assess due to CXR quality	
2. **Silhouette sign** that meets size criteria (1 rib+1 rib space) and that is associated with an opacity of any size and density a. Patient right side: yes/no/unable to assess due to CXR quality b. Patient left side: yes/no/unable to assess due to CXR quality	
3. **Pleural effusion** in the costophrenic angle or lateral pleural space that is associated with an opacity of any size and density a. Patient right side: yes/no/unable to assess due to CXR quality b. Patient left side: yes/no/unable to assess due to CXR quality	
4. **Other infiltrate** (an opacity that does not meet **Endpoint consolidation** criteria) a. Patient right side: yes/no/unable to assess due to CXR quality b. Patient left side: yes/no/unable to assess due to CXR quality	
Count and note the following:	
5. **Anterior ribs**: List the number of anterior rib ends that are totally clear of the diaphragm at any point along the diaphragm (note: lower margin of the anterior rib end must be clearly above the diaphragm): a. Patient right side: number 4–12 or unable to assess due to CXR quality b. Patient left side: number 4–12 or unable to assess due to CXR quality	
6. **Posterior ribs**: List the number of posterior rib ends that are totally clear of the mid-diaphragm (note: lower margin of the posterior rib end must be clearly above the diaphragm): a. Patient right side: number 4–12 or unable to assess due to CXR quality b. Patient left side: number 4–12 or unable to assess due to CXR quality	

### Training

**Fig. 1:**
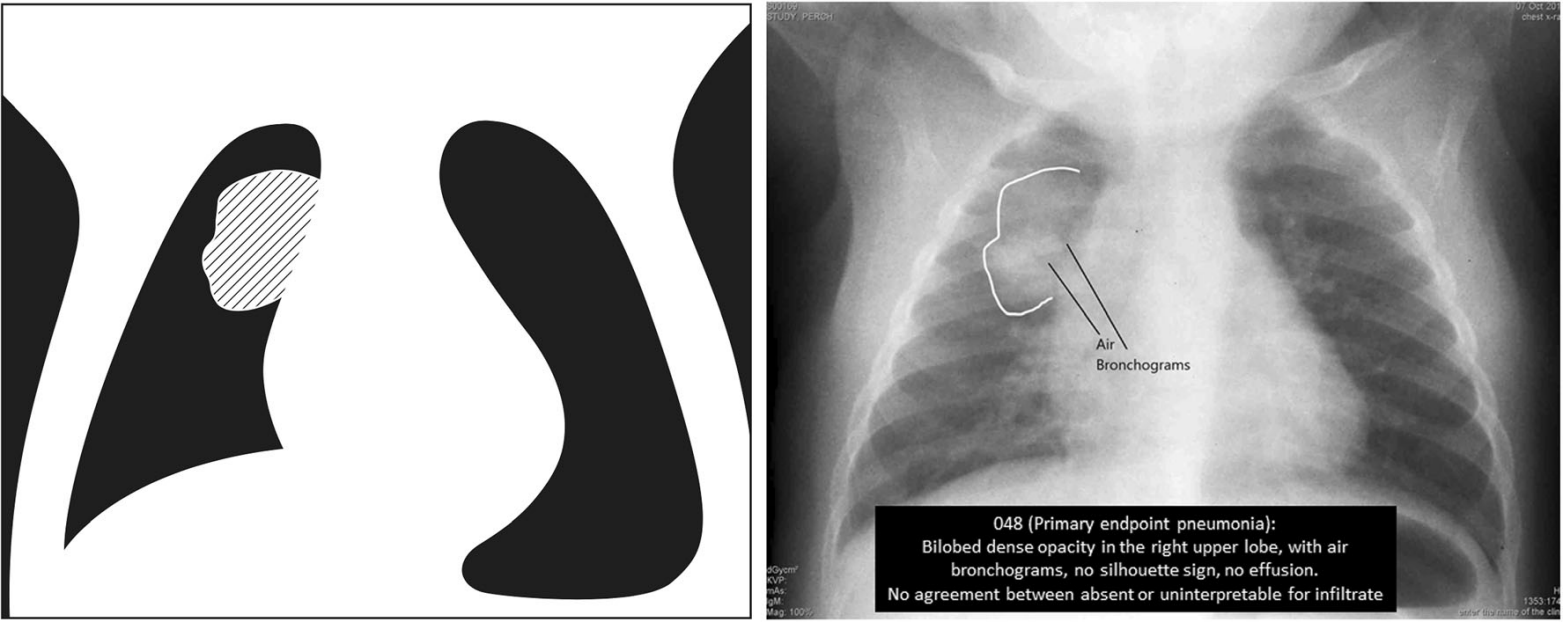
World Health Organization (WHO) Chest Radiography in Epidemiological Studies example of teaching materials for a chest radiograph with features of WHO-defined primary endpoint pneumonia

After the pretraining examination, the co-facilitators reviewed the theoretical backdrop to the WHO methodology. In addition to a more interactive, small group participatory teaching approach suitable for adults, the training content itself was designed from newer WHO Chest Radiography in Epidemiological Studies tools along with older tools dating to the original WHO chest radiograph working group. These tools included images with and without annotations that highlighted key findings on the chest radiograph image as well as cartoon drawings that simplified the key features of the image (Fig. 1). Chest radiograph images were selected to ensure representation of high- and low-quality images as well as images with a range of features meeting criteria for primary endpoint pneumonia or an other infiltrate. Importantly, similar to the pretraining test, we also restricted the pool of chest radiograph images used for teaching to only those images with findings determined to have high agreement among WHO Chest Radiography in Epidemiological Studies reading experts [17]. An exploratory component of this training included introducing an approach for participants to count anterior and posterior ribs to potentially include bilateral hyperinflation as another feature for research projects. Rib counting guidelines and teaching materials used in the training are in Table 2 and the hyperlink below. The co-facilitators introduced decision trees that were developed as reference aids for chest radiograph reading panelists to use during and after the training while interpreting radiographs (see hyperlink below). Electronic data entry systems were designed and also introduced to the participants as a part of this training, as previously described. See Table 2 for this form.

Please see https://who-cres.mcri.edu.au/resources/training-material/ for training materials developed from this work.

### Post-training examination

The meeting concluded with a post-training examination. This examination was structured similarly to the pretraining test but included a different set of 60 chest radiograph images for interpretation. Similar to the pretraining examination, the images were also selected from a pool of radiographs interpreted by the WHO Chest Radiography in Epidemiological Studies project and original WHO working group as having high agreement for the presence or absence of primary endpoint pneumonia. Participants were required to correctly interpret at least 80% of the images based on WHO Chest Radiography in Epidemiological Studies criteria to reach certification and proceed to interpreting images for the research projects. Interpretations were considered correct if the participant’s interpretation matched the reference panel’s interpretation for the binary presence or absence of primary endpoint pneumonia or whether the image was uninterpretable. Participants were not required to correctly interpret other infiltrate, as this classification historically has had high discordance among readers and was not a goal of either research project. The counting of anterior and posterior ribs was not mandated and participant certification did not consider it. All participants received a certificate of participation at the conclusion of the meeting. Participants who also met the training certification requirement were mailed a certificate noting this achievement after all of the post-training examinations were graded.

### Research plan

We assessed training participant performance on the pre- and post-training examinations. Our main outcomes of interest were the average change in test score between the pre- and post-test examinations, and the proportion of participants who correctly identified primary endpoint pneumonia (present versus absent) in ≥80% of test images. Primary endpoint pneumonia is the radiographic conclusion of interest for the associated research projects and the endpoint with good inter-rater reliability in previous studies. As we have described, the primary endpoint pneumonia and uninterpretable conclusions were constructed from coded logic. We were also interested in whether results differed between cadres (radiologist or pediatrician). Secondary outcomes included correctly identifying sub-features of primary endpoint pneumonia such as pleural effusion, silhouette sign, and endpoint consolidation, as well as the classification of other infiltrate and rib counting (anterior and posterior ribs). The ≥80% passing threshold was determined a priori and was consistent with WHO Chest Radiography in Epidemiological Studies recommendations.

We used paired *t*-tests to evaluate the difference in mean participant scores between the pre- and post-training examinations both overall and within cadres and the Student’s *t*-test to assess for any difference in the average percentage point change from pre- to post-examination results between radiologist and pediatrician participants. The McNemar chi-square test was used to test for any differences between pre- and post-test examinations in the proportion of participants receiving passing scores of ≥80% both overall and within cadres. The Pearson chi-square test was used to evaluate any differences between cadres in the percentage of participants receiving passing scores of ≥80% overall on the pretraining examination and on the post-training examination. All statistical analyses were performed using SAS (version 9.4; SAS Institute, Inc., Cary, NC).

## Results

Twenty-six of the 29 participants (89.7%) completed both the pre- and post-training examinations and were included in the analyses. Although both tests originally had 60 images, less than 10% of participants correctly classified one image from the pretraining and two images from the post-training tests. These images were considered problematic post hoc and excluded from scoring. Eleven pediatricians and 4 radiologists provided information on their work experience, with pediatricians reporting an average of 15 years and radiologists 14 years of experience interpreting chest radiographs.

### Pretraining examination

**Fig. 2:**
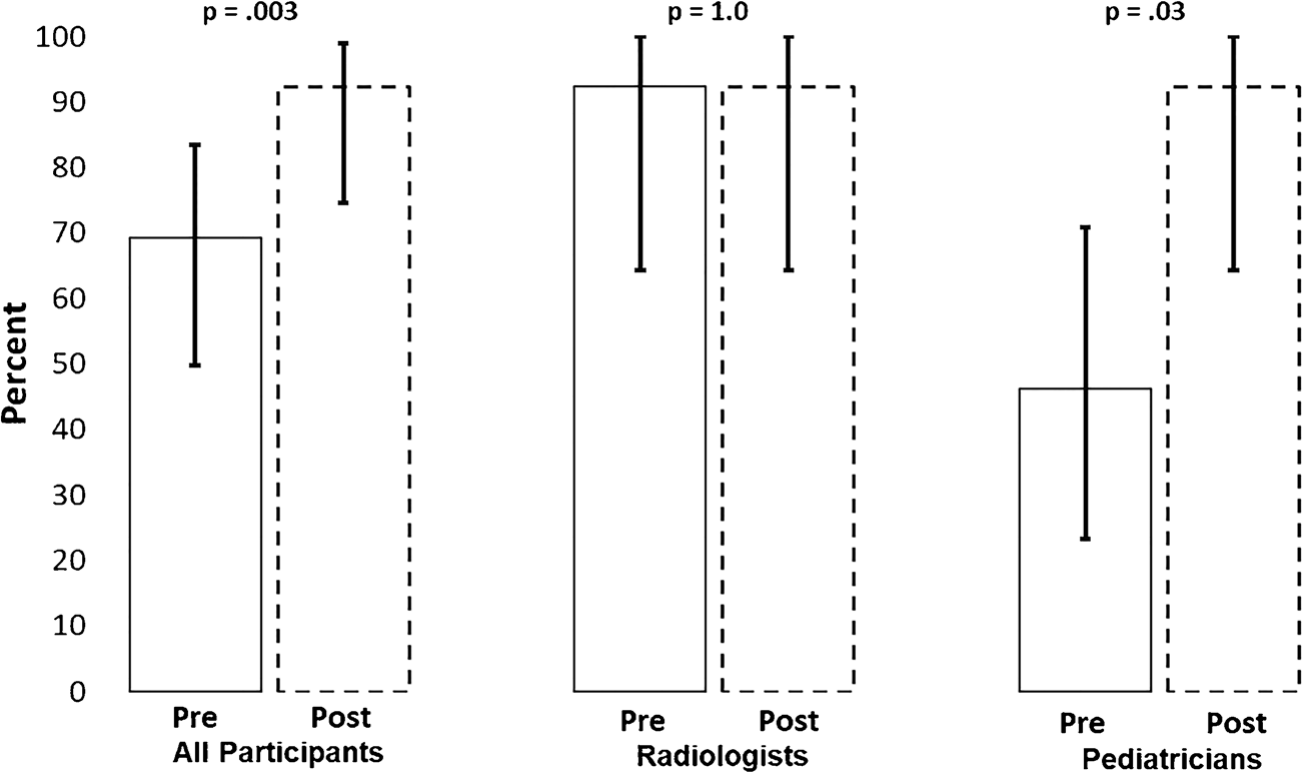
Percent of participants correctly identifying the presence/absence of primary endpoint pneumonia in ≥80% of images on the pre- and post-tests. Bars represent 95% confidence intervals

**Fig. 3:**
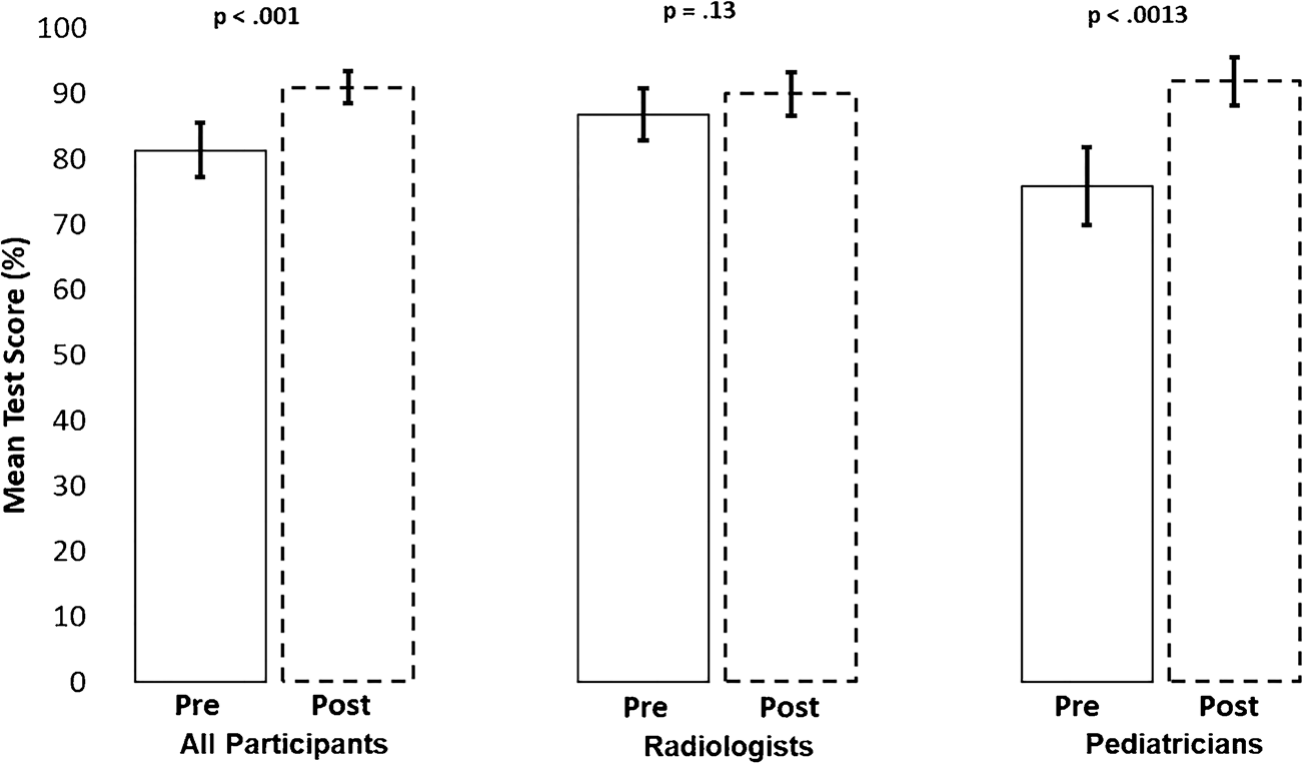
Mean pre- and post-test scores and 95% confidence intervals (*bars*) for the correct identification of the presence or absence of primary endpoint pneumonia

**Fig. 4:**
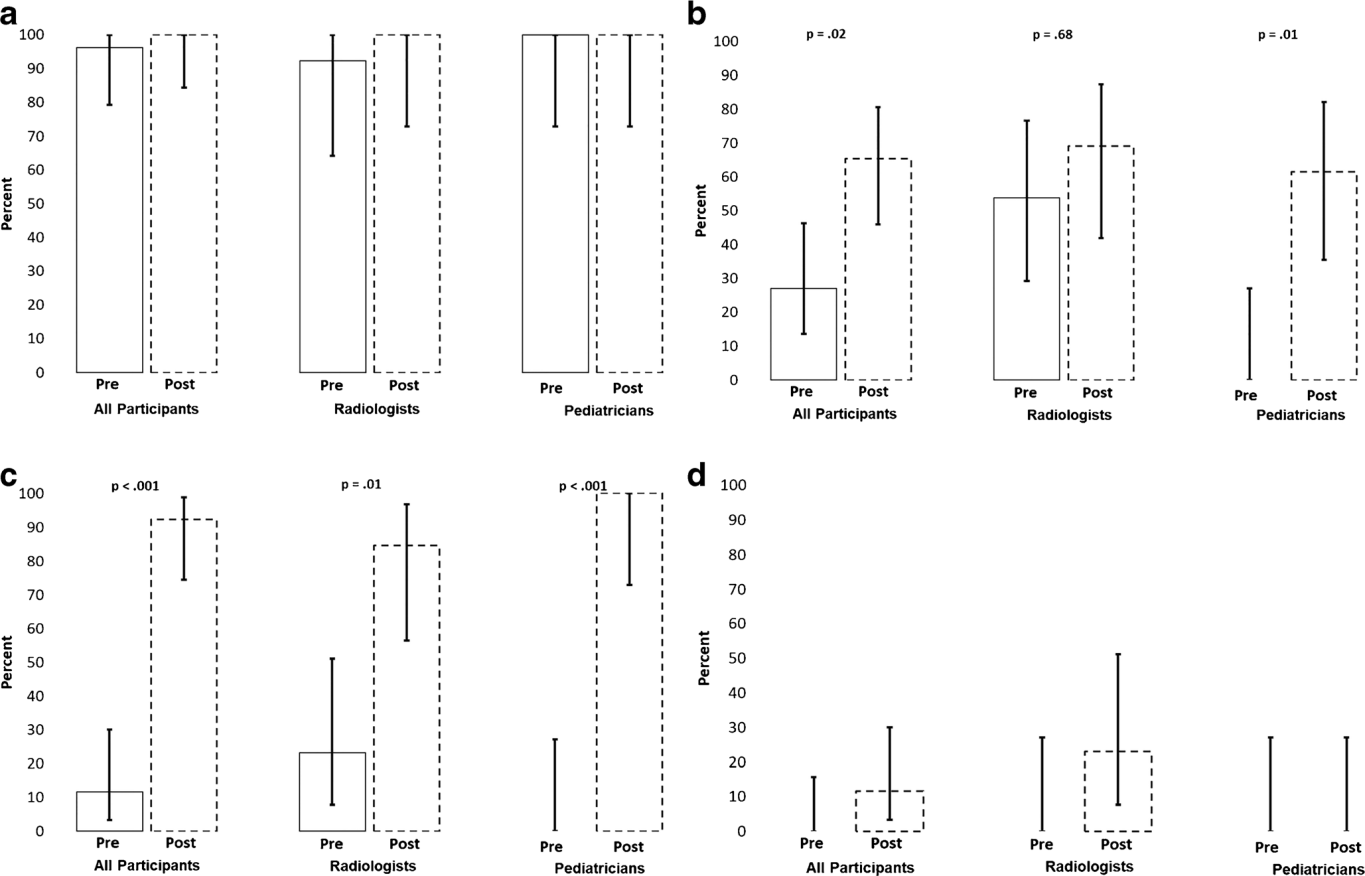
Percent of participants correctly identifying the presence/absence of pleural effusion (**a**), silhouette sign (**b**), endpoint consolidation (**c**) and other infiltrate (**d**) in ≥80% of images on the pre- and post-test. Bars represent 95% confidence intervals. McNemar tests were not performed for pleural effusion (**a**) and other infiltrate (**d**) due to small numbers in discordant cells among all groups

**Fig. 5:**
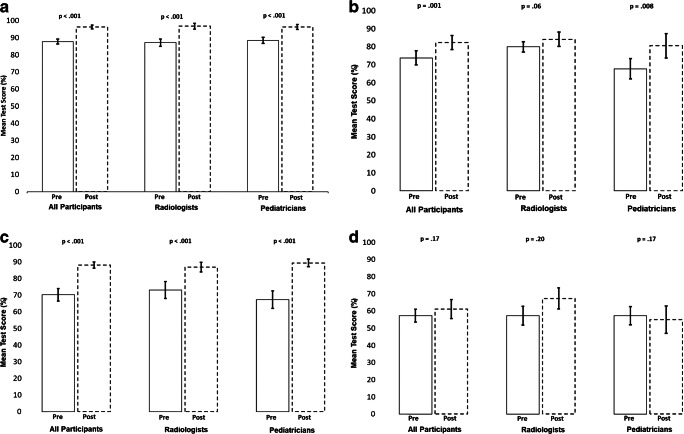
Mean pre- and post-test scores and 95% confidence intervals (*bars*) for the correct identification of the presence or absence of pleural effusion (**a**), silhouette sign (**b**), endpoint consolidation (**c**) and other infiltrate (**d**)

Overall, 69.2% (18/26) of the trainees achieved a score of 80% or higher on the pretraining examination (Fig. 2). Only 46.2% (6/13) of the pediatricians achieved 80% or higher on the pretest, compared to 92.3% (12/13) of the radiologists (*P*=0.01). The average pretraining score was 81.4% (standard deviation [SD] 10.7%) overall, with pediatricians achieving a mean score 10.8 percentage points lower compared to radiologists (75.9% [SD 10.9%] vs. 86.8% [SD 7.3%]; *P*=0.006) (Fig. 3, Online Supplementary Material 1). Scores for pleural effusion, silhouette sign, endpoint consolidation and other infiltrate are summarized in Figs. 4 and 5. More than 90% of participants overall and within cadres correctly classified ≥80% of images as having or not having pleural effusion (Fig. 4). On the other hand, both cadres did not perform as well classifying silhouette sign, endpoint consolidation or other infiltrate. No pediatricians classified ≥80% of images correctly for these features (Fig. 4). Mean pretest training scores were significantly different between radiologists (79.9% [95% confidence interval (CI): 77.1–82.7%]) and pediatricians (67.7% [95% CI: 62.0–73.3%]) for silhouette sign, but did not differ for other findings.

### Post-training examination

On the post-training examination, 92.3% (24/26) of all trainees scored 80% or higher (Fig. 2). The mean percentage of images classified correctly by all participants on the post-training examination was 90.9% (SD 6.4%). There was no statistical difference in the mean post-training score between the groups for any findings. Four of the 26 participants regressed on the post-training examination, with 2 falling below the 80% threshold.

### Pretraining versus post-training examinations

The percentage of participants passing the examination increased from 69.2% on the pretraining test to 92.3% on the post-training test (*P*=0.003) (Fig. 2). This improvement was attributed to a large increase (46.2 percentage points) in the proportion of pediatricians passing the post-training examination compared to the pretraining examination (*P*=0.03). Among all participants, the average score improved by 9.6 percentage points (95% CI: 5.0–14.1 percentage points) between the pre- and the post-training tests (*P*<0.001) (Fig. 3, Online Supplementary Material 1). Although the mean radiologist score did not statistically improve between the pre- and post-training test (*P*=0.13), the mean pediatrician score increased by 16.0 percentage points (95% CI: 9.3–22.7 percentage points; *P*<0.001). The proportion of participants correctly classifying ≥80% of images for endpoint consolidation increased by 80.8 percentage points between the pre- and post-training tests (*P*<0.001) (Fig. 4). A similar pattern of improvement was observed within both cadres. In addition, among the 8 participants who scored below 80% on the pretraining examination, 7 (87.5%) passed the post-training examination. While the proportion of participants classifying ≥80% of radiographs for silhouette sign also increased, this improvement was statistically significant among pediatricians only (0% vs. 61.5%, *P*=0.01). The mean examination score improved overall and within both cadres for pleural effusion and endpoint consolidation, but only for pediatricians for silhouette sign (Fig. 5). No statistically significant changes between the pre- and post-training tests for other infiltrate were observed for both the percentage of participants passing the examinations and the mean score, both overall and within cadres.

Please see Online Supplementary Material 2 for test results for rib counting overall. Test results for rib counting by participant are available in Online Supplementary Material 3 (anterior ribs), 4 (posterior ribs) and 5 (overall).

## Discussion

We developed a training course for the WHO standardized interpretations of pediatric chest radiographs to support two research studies in India. Our findings show significant improvements in the correct identification of endpoint consolidation and silhouette sign, both key features of the radiologic definition of primary endpoint pneumonia, that have not been reported previously. Following recommendations from the WHO Chest Radiography in Epidemiological Studies project, we considered readers to have been successfully standardized to the radiologic definitions if they correctly identified the presence or absence of primary endpoint pneumonia in ≥80% of images. While more radiologists (12/13) achieved this standard at baseline compared to pediatricians (6/13), after training the same number of pediatricians and radiologists achieved the standard (both 12/13). Similarly, radiologists had better pretraining mean scores for primary endpoint pneumonia and silhouette sign when compared to pediatricians, but this difference did not persist in test scores after training. These findings suggest that future trainings should consider focusing teaching and standardization efforts on endpoint consolidation and silhouette sign, with an emphasis on the objective measurement approaches for both, rather than primary endpoint pneumonia more generally, as has been done previously.

Over a period of two decades, the WHO pediatric chest radiograph interpretation methodology has been used for landmark pediatric research, including efficacy and effectiveness studies of bacterial conjugate vaccines and lower respiratory infection epidemiology research in low-income and middle-income countries [7, 9, 11–13, 16]. The results of this research have influenced global policy on lower respiratory infection vaccine introduction in low-income and middle-income countries as well as pediatric lower respiratory infection treatment guidelines. Recently, the WHO Chest Radiography in Epidemiological Studies working group refined this methodology and introduced a contemporary set of teaching tools that includes several hundred chest radiograph images interpreted by experts, categorized by level of agreement and annotated for educational purposes [17]. To elevate the rigor of research applying this methodology, the WHO Chest Radiography in Epidemiological Studies working group recommended such research should evaluate and report the effectiveness of the chest radiograph reading panel training, in addition to reporting the ongoing performance of the panel during implemented research [17]. Our findings show that our training, which applied and adapted the WHO Chest Radiography in Epidemiological Studies images and educational tools, was successful. More than 90% of participants passed the post-training certification examination, average overall post-training test scores improved compared to pretraining scores in nearly all areas, and the pretraining test result differences between radiologists and pediatricians disappeared after both groups were similarly trained.

Research has largely focused on analyzing the performance of reading panels applying the WHO chest radiograph methods during a research project rather than on any training methodology itself. The original WHO Radiology Working Group included 20 participants, a mix of pediatricians and radiologists, who developed and then applied the interpretation method to 222 chest radiographs to generate reference interpretations [8]. The Pneumonia Etiology Research for Child Health (PERCH) study described the training of a reading panel of 18 physicians (9 radiologists and 9 pediatricians) but did not report the effectiveness of the training [20]. In PERCH, all readers participated in a two-day in-person training [20]. At the conclusion of the training, the readers completed a post-training examination of 20 reference images randomly selected from a pool of 222 WHO Radiology Working Group reference images previously described [8, 20]. To be certified, the readers were required to correctly classify at least 50% of all 20 images, including ≥66% of images with primary endpoint pneumonia and ≥66% of images that did not have an endpoint consolidation, other infiltrate or pleural effusion [20]. If readers did not achieve these thresholds, they were remediated and evaluated with a different set of 20 images until the thresholds were met [20]. A recent pneumococcal conjugate vaccine effectiveness study from Bangladesh described a two-day training of an eight-member reading panel (six radiologists and two pediatricians) but did not report the effectiveness of the training [10]. The authors additionally described and reported on semiannual re-standardization trainings and additional quality control procedures applied during the study [10]. Similar to PERCH, this Bangladesh pneumococcal conjugate vaccine effectiveness study analyzed the performance of the reading panel during the study [10, 20]. In order to build on this research, we both described our training and analyzed its effectiveness not only for classifying primary endpoint pneumonia but also at a more granular level for the features that inform the primary endpoint pneumonia classification (i.e. endpoint consolidation, pleural effusion, silhouette sign). Our positive results suggest that future studies incorporating the WHO chest radiograph method should consider modelling this training approach, especially the small group interactive method we employed along with objective assessments. Both helped to identify preexisting knowledge and to target individual learning styles in an effort to optimize the training.

In addition to reporting training effectiveness on endpoint consolidation, silhouette sign, pleural effusion and other infiltrate, we also stratified the performances by pediatrician and radiologist cadres. The WHO Chest Radiography in Epidemiological Studies group refined the original WHO definitions to increase the objectiveness of the endpoint consolidation and the silhouette sign features by adding measurable size dimensions [17]. Our results show that after training, the performances of pediatricians and radiologists were no different in the classification of primary endpoint pneumonia. In addition, our analyses indicate that the training taught participants to effectively apply the refined endpoint consolidation and silhouette sign definitions. Specifically, both pediatricians and radiologists improved their application of the endpoint consolidation criteria, and pediatricians — more than radiologists — improved classifying images for silhouette sign. While classification of pleural effusion improved among radiologists, performance was high at baseline. On the other hand, the training did not change the overall low performance of participants, regardless of cadre, of classifying other infiltrate. In sum, these results show that not only can the revised WHO Chest Radiography in Epidemiological Studies endpoint consolidation and silhouette sign criteria be successfully applied, but that with sufficient training, pediatricians who are unlikely to interpret chest radiographs as frequently as radiologists can learn to apply these methods as effectively as radiologists. We believe that these refined, more objective definitions for endpoint consolidation and silhouette sign will be key for improving the validity and reproducibility of research applying this methodology. These results support more objective measurement criteria for these features. Depending upon availability, future panels may be as effective when composed of non-radiologists. Future trainings may decide to place more emphasis on different radiographic features depending on the composition of their participants.

This research has several limitations. First, pediatricians and radiologists are scarce in most low-income and middle-income countries and our findings may not apply to a panel composed of non-pediatricians and non-radiologists. Similar research should be conducted on such panels and our results should be interpreted within this context. Second, the chest radiograph images used in the pre- and post-training examinations as well as during the training itself were images with high expert agreement for the classification of primary endpoint pneumonia. Images collected during research in low-income and middle-income countries will have more variable quality and agreement among reading panels. It is, therefore, key to monitor the performance of readers throughout research projects using this methodology, provide remediation as needed and conduct refresher trainings. Lastly, the WHO methodology does not apply to clinical care and any work attempting to extend this method to clinical applications is outside our intended scope.

We have several other recommendations for future reading panel trainings based on our experience. Future trainings should consider adding a midpoint assessment to better understand participant progress to allow the training to be more adaptive to learning needs. Our training was 3 days and participants universally recommended that future trainings be limited to 2 days. Other chest imaging modalities like lung ultrasound have recently been reported in pediatric research in low-income and middle-income countries and hold promise [21]. We recommend projects using newer modalities like lung ultrasound apply similar training approaches if their work includes reading panels.

## Conclusion

Results indicate that our training approach using revised WHO Chest Radiography in Epidemiological Studies definitions and adapted tools was successful. We recommend that future research using the WHO chest radiograph method or other similar imaging methods consider modelling their trainings after this approach.

## Supplementary Information

Online Supplementary Material 1 Pre- and post-test performance for the interpretation of chest radiographs by training participants for the presence or absence of primary endpoint pneumonia (DOCX 20 kb)

Supplementary Figure (PNG 101 kb)

Online Supplementary Material 2 Mean test scores and 95% confidence intervals for correct determination of rib counts. High Resolution Image (TIFF 549 mb)

Supplementary Figure (PNG 123 kb)

Online Supplementary Material 3 Test scores for anterior rib counts. High Resolution Image (TIFF 549 mb)

Supplementary Figure (PNG 215 kb)

Online Supplementary Material 4 Test scores for posterior rib counts. High Resolution Image (TIFF 549 mb)

Supplementary Figure (PNG 119 kb)

Online Supplementary Material 5 Test scores for anterior and posterior ribs (all rib counts answered correctly). High Resolution Image (TIFF 549 mb)
