# Supplementary material for: Training physicians in India to interpret pediatric chest radiographs according to World Health Organization research methodology
Source: Pediatr Radiol. 2021 Mar 11;51(8):1322–31. doi: 10.1007/s00247-021-04992-2 (PMC8266794; doi:10.1007/s00247-021-04992-2)
Supplement: Supplementary file 1 — Pre- and post-test performance for the interpretation of chest radiographs by training participants for the presence or absence of primary endpoint pneumonia (DOCX 20 kb) [file 247_2021_4992_MOESM1_ESM.docx]

**Online Supplementary Material 1**

| Participant cadre, *n*=26^a^ | Pretest score (%), *n*=59^b^ | Post-test score (%), *n*=58^b^ | Difference between pretest and post-test (percentage points) |
| --- | --- | --- | --- |
| Pediatrician | 81.4 | 100 | +18.6 |
| Pediatrician | 86.4 | 100 | +13.6 |
| Radiologist | 83.1 | 89.7 | +6.6 |
| Radiologist | 88.1 | 89.7 | +1.5 |
| Pediatrician | 62.7 | 86.2 | +23.5 |
| Radiologist | 96.6 | 96.6 | −0.1 |
| Pediatrician | 76.3 | 96.6 | +20.3 |
| Pediatrician | 59.3 | 94.8 | +35.5 |
| Pediatrician | 78.0 | 75.9 | −2.1 |
| Pediatrician | 83.1 | 98.3 | +15.2 |
| Radiologist | 88.1 | 100 | +11.9 |
| Radiologist | 94.9 | 89.7 | −5.3 |
| Radiologist | 94.9 | 94.8 | −0.1 |
| Radiologist | 93.2 | 94.8 | +1.6 |
| Pediatrician | 86.4 | 96.6 | +10.1 |
| Radiologist | 86.4 | 89.7 | +3.2 |
| Pediatrician | 88.1 | 91.4 | +3.2 |
| Pediatrician | 83.1 | 87.9 | +4.9 |
| Radiologist | 83.1 | 91.4 | +8.3 |
| Radiologist | 69.5 | 87.9 | +18.4 |
| Radiologist | 81.4 | 84.5 | +3.1 |
| Radiologist | 83.1 | 77.6 | −5.5 |
| Pediatrician | 55.9 | 87.9 | +32.0 |
| Pediatrician | 78.0 | 87.9 | +10.0 |
| Radiologist | 86.4 | 82.8 | −3.7 |
| Pediatrician | 67.8 | 91.4 | +23.6 |
| Mean pediatrician score (SD)/mean difference (95% CI) | 75.9 (10.9) | 91.9 (6.8) | +16.0 (9.3, 22.7) |
| Mean radiologist score (SD)/mean difference (95% CI) | 86.8 (7.3) | 89.9 (6.0) | +3.1 (−1.0, 7.2) |
| Mean total score (SD)/mean difference (95% CI) | 81.4 (10.7) | 90.9 (6.4) | +9.6 (5.0, 14.1) |

^a^ 3 participants were excluded because they did not complete both the pretest and post-test

^b^ Original tests had 60 images. One image from the pretest and two images from the post-test proved to be poor test images and problematic for almost all participants, and thus were excluded from scoring post hoc

*CI* confidence interval, *SD* standard deviation
