# Supplementary figures and images for: Training physicians in India to interpret pediatric chest radiographs according to World Health Organization research methodology
Source: Pediatr Radiol. 2021 Mar 11;51(8):1322–31. doi: 10.1007/s00247-021-04992-2 (PMC8266794; doi:10.1007/s00247-021-04992-2)

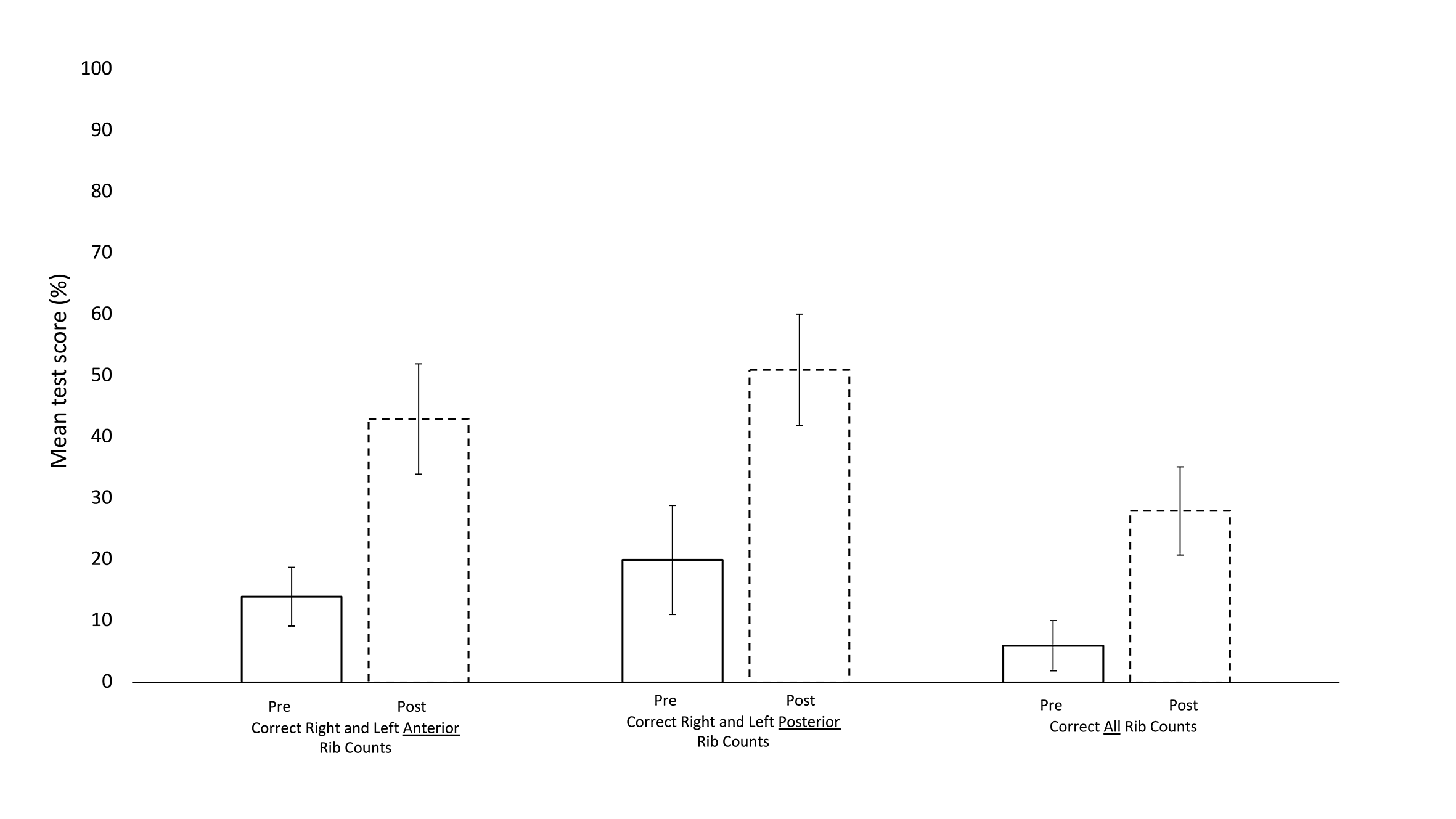

Supplement: Supplementary file 2 — (PNG 101 kb) [file 247_2021_4992_Fig6_ESM.png]

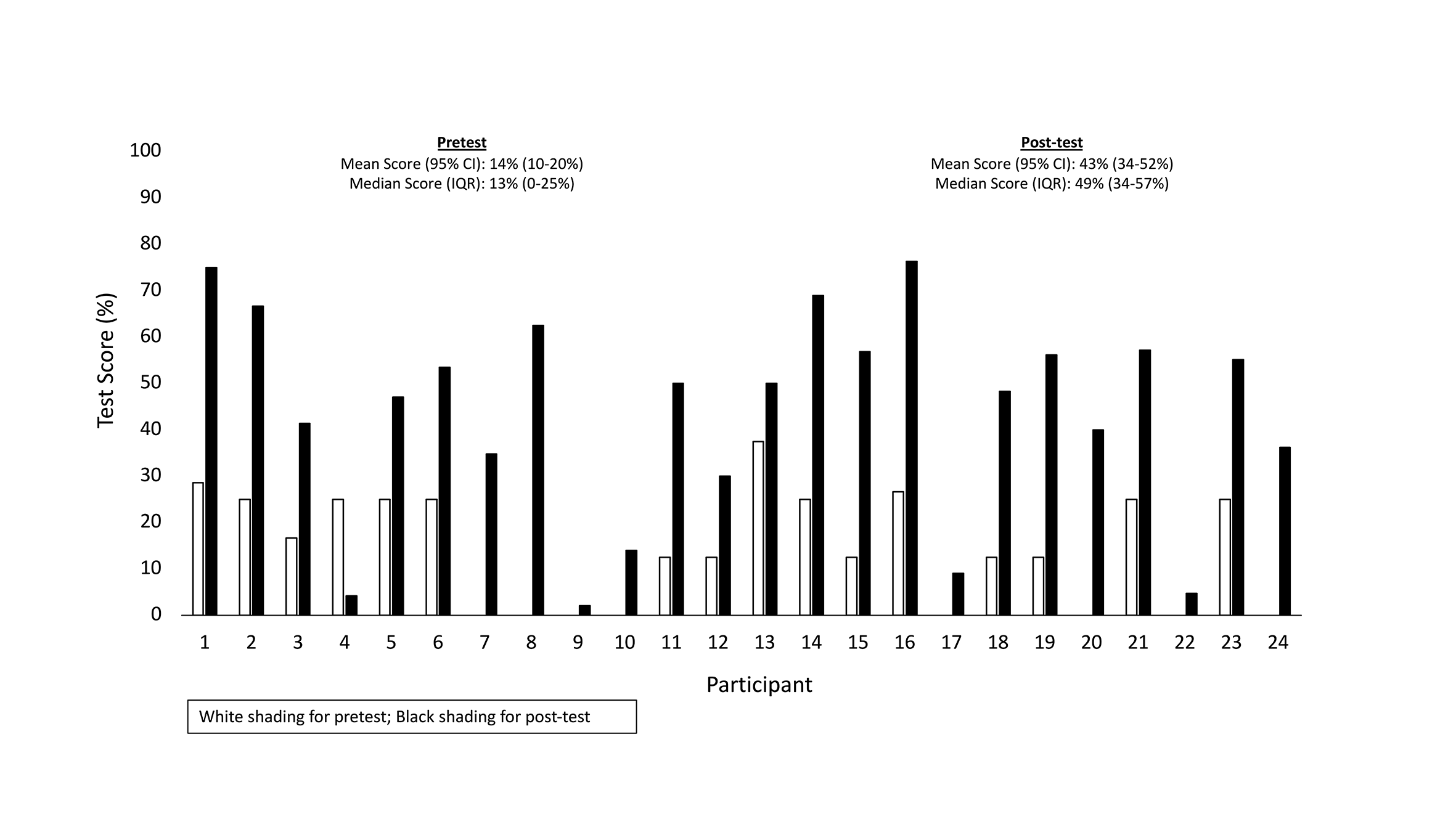

Supplement: Supplementary file 4 — (PNG 123 kb) [file 247_2021_4992_Fig7_ESM.png]

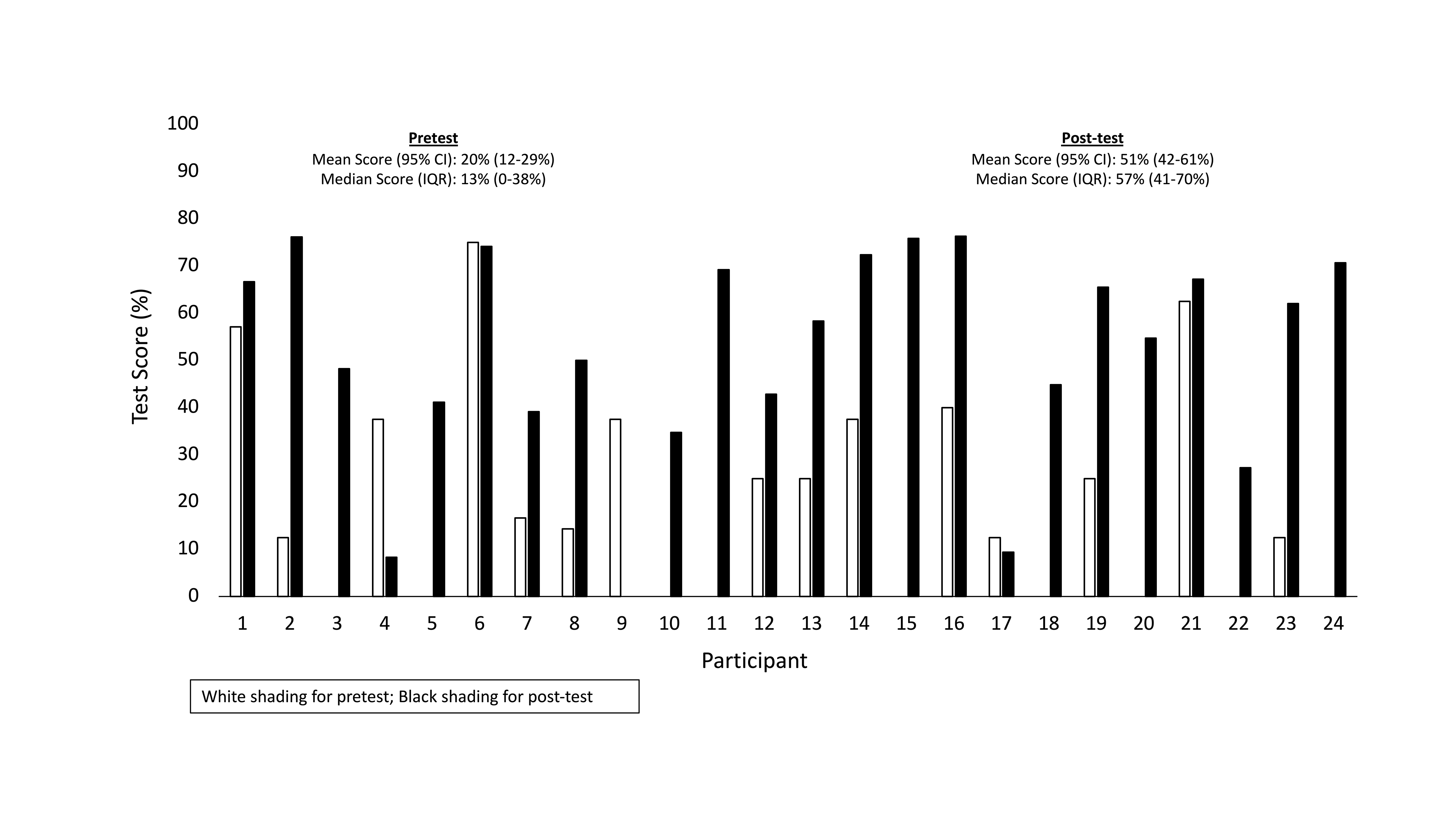

Supplement: Supplementary file 6 — (PNG 215 kb) [file 247_2021_4992_Fig8_ESM.png]

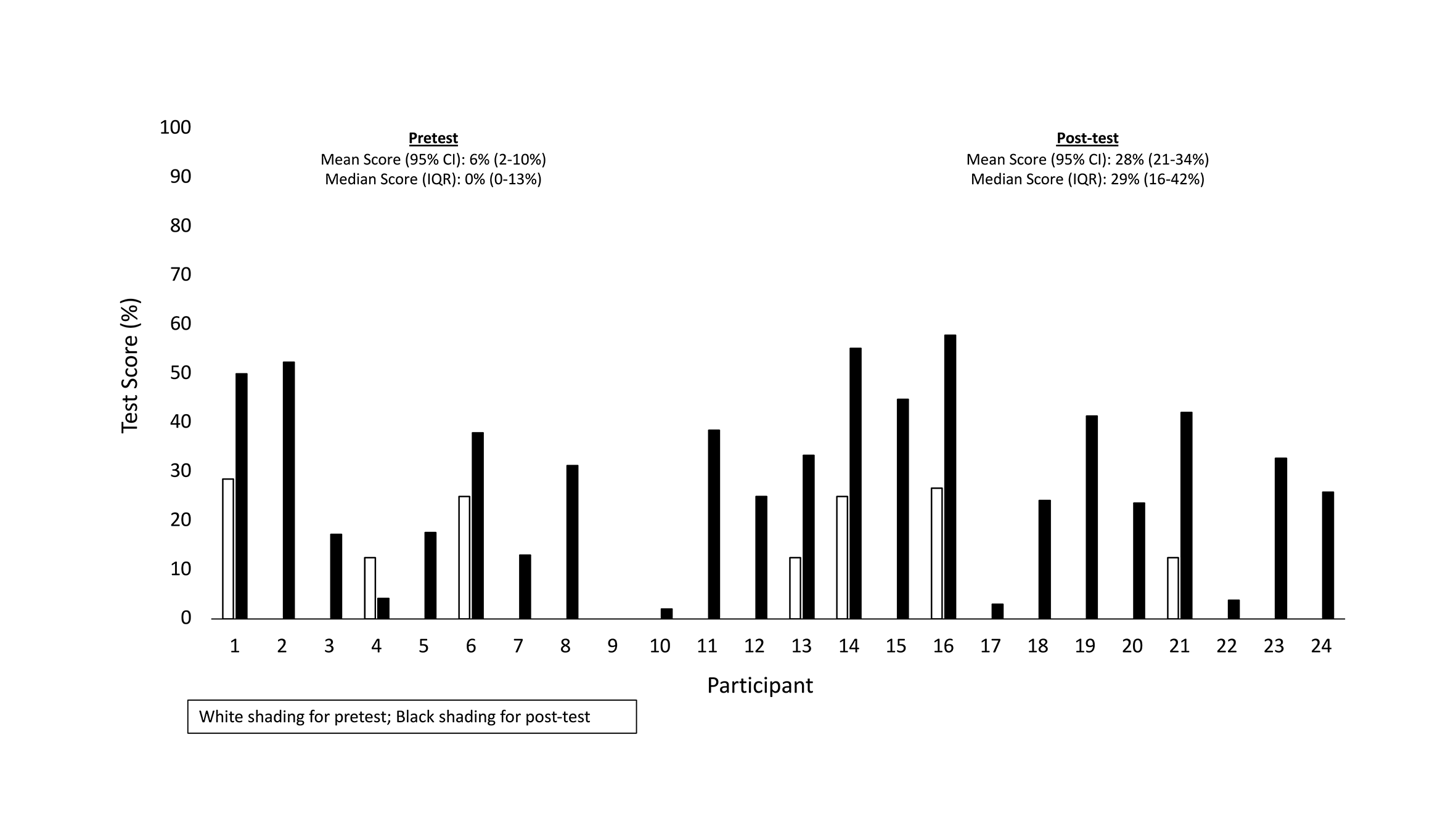

Supplement: Supplementary file 8 — (PNG 119 kb) [file 247_2021_4992_Fig9_ESM.png]
